# Platelet-Rich Fibrin-Enhanced Fat Grafting for Gluteal Contour Deformities in Compromised Soft Tissue: A Technical Note

**DOI:** 10.7759/cureus.115938

**Published:** 2026-09-08

**Authors:** Oscar A Vazquez, Taryn Gilgallon, Kevin Abadi, Hilton Becker

**Affiliations:** 1 Surgery, University of Florida, Gainesville, USA; 2 Medicine, Florida International University, Miami, USA; 3 Emergency Medicine, Florida Atlantic University, Boca Raton, USA; 4 Surgery, Florida Atlantic University, Boca Raton, USA

**Keywords:** dermatology, fat grafting, gluteal autologous fat grafting, plastic surgery, platelet rich fibrin, prf

## Abstract

Gluteal contour deformities resulting from localized soft tissue atrophy represent a challenging reconstructive problem, particularly in patients with compromised recipient-site tissue quality. Autologous fat grafting is commonly used for contour restoration, although graft retention may be variable. We describe a technical approach to platelet-rich fibrin (PRF)-enhanced autologous fat grafting that incorporates mechanical micronization of PRF, integration with harvested adipose tissue, and micro-aliquot injection. A representative case of steroid injection-induced gluteal lipoatrophy is presented to demonstrate the application of the technique and long-term clinical outcomes. PRF was prepared from two 10-mL red-top Vacutainer tubes without anticoagulant using low-speed centrifugation at 2800 rpm for five minutes. The resulting fibrin clot was mechanically micronized and combined with harvested adipose tissue at a 1:3 PRF-to-fat ratio by volume. A total of approximately 20 mL of composite graft was injected into the affected gluteal region within the subcutaneous plane. The patient demonstrated sustained clinical improvement in contour without clinically apparent overcorrection or recurrent contour deformity at 5-year follow-up. Because this report describes a single case without a control group, objective volumetric assessment, or imaging-based evaluation, the observed durability cannot be attributed specifically to PRF augmentation. This technical report demonstrates the feasibility of incorporating mechanically micronized PRF into structural fat grafting and provides long-term clinical observation following its use in a compromised gluteal soft tissue bed. Further controlled studies are required to determine whether PRF augmentation provides an incremental benefit over conventional fat grafting alone.

## Introduction

Gluteal contour deformities secondary to localized soft tissue atrophy represent a persistent challenge in aesthetic and reconstructive surgery. These deformities may arise from trauma, prior procedures, or iatrogenic causes such as corticosteroid injection. [1-3] In addition to localized volume loss, compromised tissue quality may further limit reconstructive options and affect the predictability of contour restoration. Autologous fat grafting has become an established approach for soft tissue contour restoration. However, the degree of graft retention may vary because of differences in recipient-site vascularity, graft handling, injection technique, and individual biological factors [4,5]. These considerations may be particularly relevant in areas of pre-existing tissue atrophy or altered soft tissue quality.

Platelet-rich fibrin (PRF) is a second-generation autologous platelet concentrate characterized by a fibrin matrix capable of sustained release of biologically active mediators, including vascular endothelial growth factor (VEGF). Unlike platelet-rich plasma (PRP), PRF is prepared without an anticoagulant, allowing the formation of a fibrin matrix during processing. Experimental studies have investigated PRF as an adjunct to fat grafting because of its potential effects on angiogenesis, adipogenesis, apoptosis, and extracellular matrix remodeling [6-9]. Mechanical micronization of the fibrin matrix permits incorporation of PRF into an injectable graft, and previous work has described PRF-enhanced fat grafting using this approach [10,11].

This report does not aim to establish that PRF improves fat graft survival or volumetric retention. Rather, it describes the technical integration of mechanically micronized PRF into a structural fat grafting workflow and its application to steroid injection-induced gluteal lipoatrophy in a compromised soft tissue bed. The technical contribution includes the preparation and micronization of PRF, its incorporation with harvested adipose tissue at a defined 1:3 PRF-to-fat ratio, and delivery using small-volume aliquots through a blunt cannula in the subcutaneous plane. Five-year clinical observation is presented to document the long-term clinical appearance following the overall procedure. The case is intended to demonstrate technical feasibility and clinical application rather than comparative efficacy.

Although PRF-enhanced fat grafting has been previously described, including the use of micronized PRF to facilitate injection, the present report focuses specifically on its integration into a structural fat grafting protocol for steroid injection-induced gluteal lipoatrophy. The principal contribution is therefore the description of this technical workflow in a compromised gluteal recipient bed together with five-year clinical observation, rather than the introduction of PRF as a novel adjunct to fat grafting itself [10,11].

## Technical report

Patient selection

Candidates for the described technique are selected based on the presence of a localized contour deformity, clinical evidence of soft tissue atrophy or compromised tissue quality, and sufficient adipose tissue at the donor site. Patient selection should also consider the extent of the deformity, the quality of the recipient tissue, the availability of suitable donor tissue, and the patient’s expectations for contour restoration.

Tumescent infiltration

Donor sites are infiltrated with Klein's solution to facilitate local anesthesia, vasoconstriction, and hydrodissection.

Fat harvest

Adipose tissue is harvested using low-pressure lipoaspiration techniques intended to preserve adipocyte viability. Care is taken to minimize shear stress and traumatic manipulation, consistent with established principles of structural fat grafting [4,5].

PRF preparation

PRF is prepared from peripheral venous blood collected into two 10-mL red-top Vacutainer tubes. No anticoagulant is added. The absence of anticoagulant permits the formation of a fibrin matrix, which distinguishes PRF from PRP preparations. The blood is subjected to low-speed centrifugation at 2800 rpm for five minutes. Because the centrifuge model and rotor characteristics used for the historical procedure were not recorded, the centrifugation protocol is reported in revolutions per minute and duration rather than converted to relative centrifugal force (RCF). This limitation is acknowledged to facilitate accurate reporting of the procedure without introducing an estimated or reconstructed RCF.

Following centrifugation, the fibrin clot is separated from the residual red blood cell fraction and discarded blood components. The fibrin clot is initially reduced in size using Metzenbaum scissors and subsequently mechanically micronized using a micronizer. Mechanical micronization converts the fibrin clot into a form that can be incorporated into an injectable composite graft. Alternative methods of mechanical micronization may also be used [10,11].

Fat-PRF integration

Harvested adipose tissue is combined with mechanically micronized PRF to create a composite graft. For the presented case, approximately 5 mL of PRF was combined with 15 mL of harvested adipose tissue, corresponding to a 1:3 PRF-to-fat ratio by volume. The resulting composite graft had a total volume of approximately 20 mL. The composite graft was gently mixed to promote relatively uniform distribution of the micronized PRF throughout the adipose tissue while minimizing unnecessary mechanical disruption. The composite graft was placed in a 20-mL syringe and subsequently transferred to a 1-mL syringe for injection through a blunt 1-mm cannula.

Previous experimental work has investigated PRF concentration and mixing ratios in relation to fat graft outcomes [8]. The 1:3 ratio used in this case was the operative mixing ratio and was not prospectively optimized; therefore, the present report does not establish this ratio as an optimal concentration.

Injection technique

The composite graft is injected using multiple passes and small-volume aliquots within the subcutaneous plane. The cannula is maintained within the subcutaneous tissue during graft deposition, with controlled micro-aliquot delivery used to distribute the composite graft throughout the area of contour deficiency. In the presented case, all graft placement was strictly confined to the subcutaneous plane. No intramuscular or deeper-plane injection was performed. The blunt 1-mm cannula was used to distribute the composite graft through multiple passes while avoiding penetration into deeper tissues.

Postoperative management

Postoperative care includes avoidance of direct pressure on the grafted area, gradual return to normal activity, and serial clinical evaluation of contour and volume retention.

Clinical application

**Figure 1:**
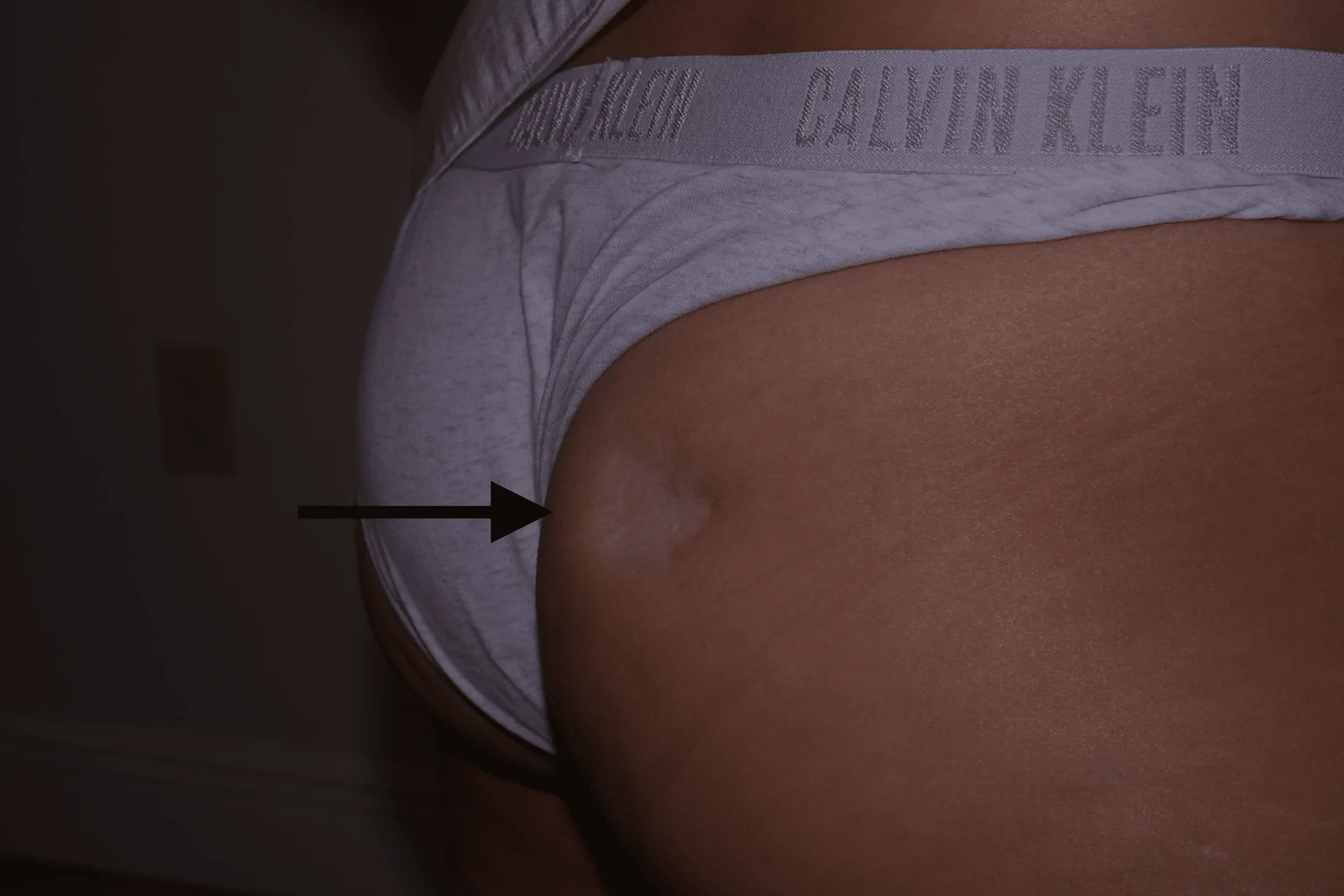
Preoperative photograph of a 25-year-old woman demonstrating right gluteal contour deformity with localized volume loss following corticosteroid injection

A 25-year-old woman presented with a right gluteal contour deformity following an intramuscular corticosteroid injection administered for pityriasis rosea. Examination demonstrated localized volume loss with associated skin changes consistent with soft tissue atrophy (Figure 1).

**Figure 2:**
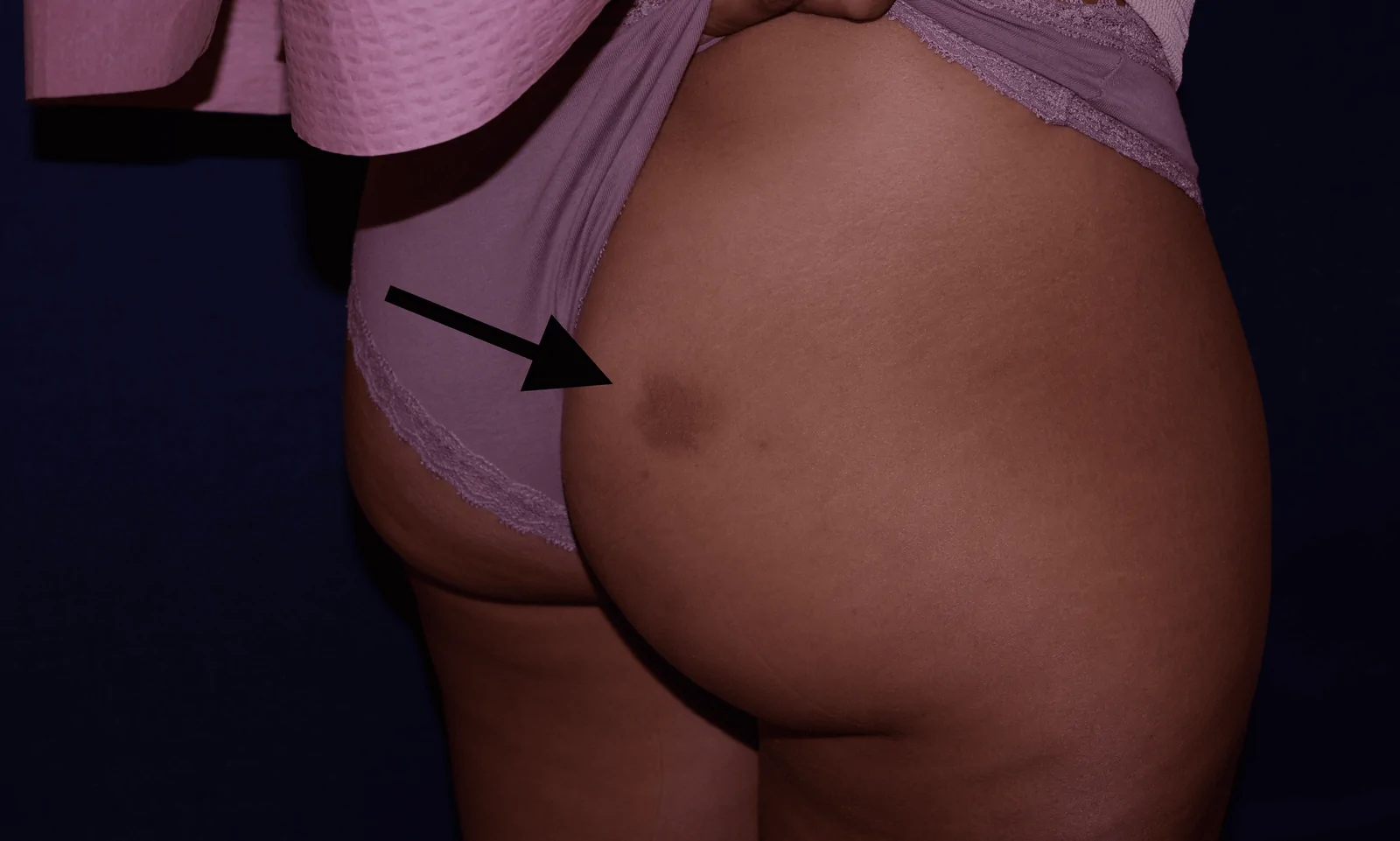
One-month postoperative photograph demonstrating clinical improvement in the gluteal contour deformity

**Figure 3:**
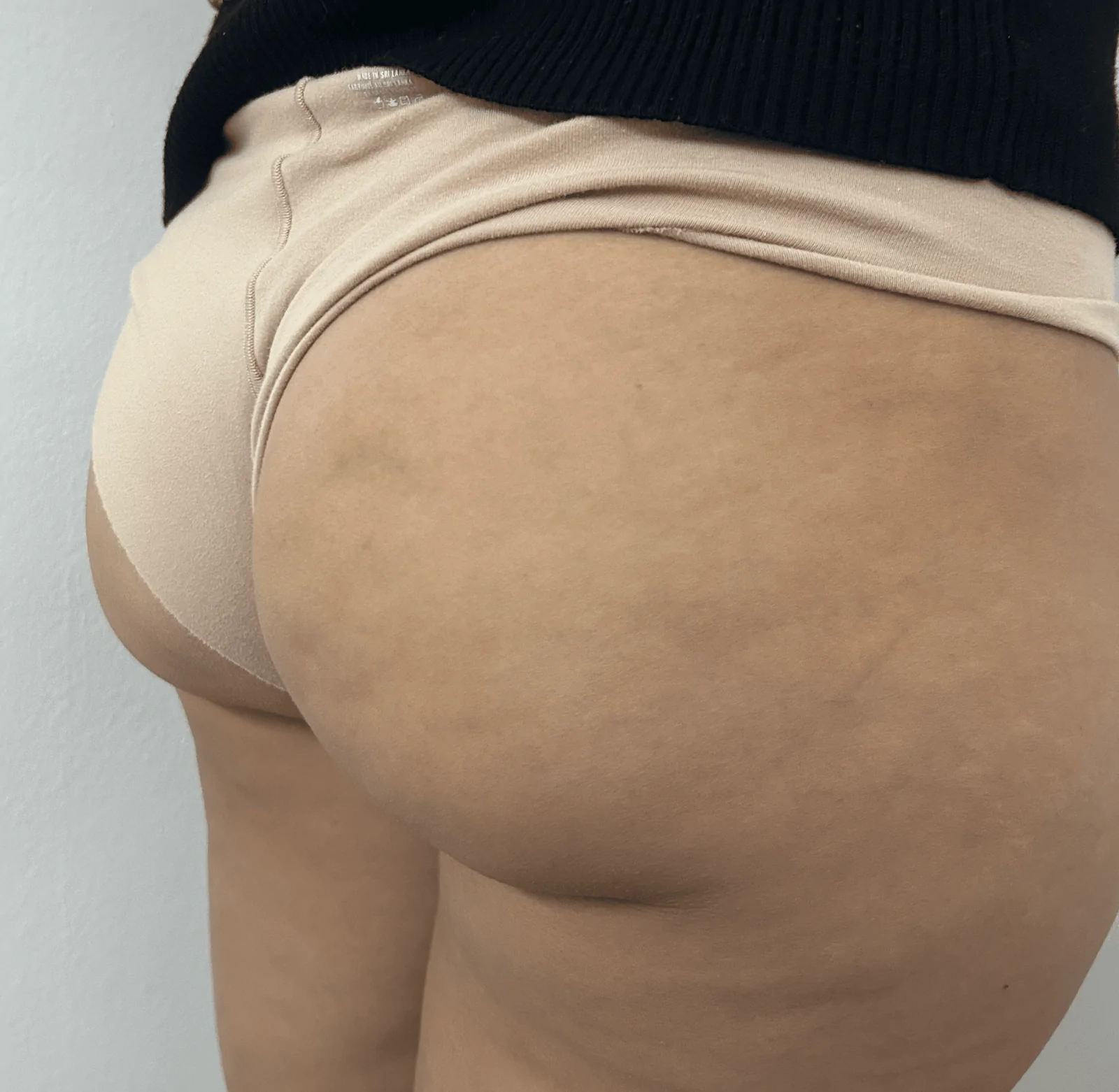
Five-year postoperative photograph demonstrating maintained clinical contour correction and stable external appearance

The patient underwent PRF-enhanced autologous fat grafting using the technique described above. The procedure was performed under local anesthesia with lipoaspiration of the lower back. Approximately 20 mL of composite graft, consisting of 5 mL of mechanically micronized PRF and 15 mL of harvested adipose tissue (1:3 PRF-to-fat ratio by volume), was injected into the affected gluteal region. All graft placement was performed strictly within the subcutaneous plane, with no intramuscular or deeper-plane injection. At one-month follow-up, clinical improvement in the gluteal contour deformity was observed (Figure 2). Figure 3 shows the postoperative photograph at five years.

## Discussion

Optimization of fat graft survival remains an important consideration in aesthetic and reconstructive surgery. Conventional structural fat grafting emphasizes atraumatic harvest, careful handling, and deposition of small-volume aliquots to maximize contact between grafted adipose tissue and the recipient bed [4,5]. Biologic augmentation strategies, including platelet concentrates such as PRF, have subsequently been investigated as potential adjuncts to these established techniques.

PRF differs from PRP in its preparation and structural characteristics. PRF is produced without anticoagulant, allowing the formation of a fibrin matrix that can serve as a scaffold for the sustained release of biologically active mediators. Experimental and clinical studies have reported biological effects associated with PRF augmentation, including effects on angiogenesis, adipogenesis, apoptosis, collagen production, and measures of fat graft outcome [6-9]. These findings provide a biological rationale for investigating PRF as an adjunct to fat grafting, particularly in recipient sites where tissue quality or vascularity may be compromised. However, evidence from prior studies does not establish that PRF was responsible for the clinical outcome observed in the present patient.

The present technical report describes the incorporation of mechanically micronized PRF into a structural fat grafting workflow for steroid injection-induced gluteal lipoatrophy. Mechanical micronization permits the reduction of the fibrin matrix into an injectable form that can be distributed throughout the adipose graft [10,11]. The technique is combined with established structural fat grafting principles, including atraumatic fat harvest, gentle graft handling, small-volume aliquot deposition, and controlled delivery through a blunt cannula. These technical elements are intended to facilitate graft handling and recipient-tissue contact rather than to establish a specific biological effect of PRF.

The use of the micronizer is also relevant to the technical workflow because the device was designed to facilitate mechanical micronization of tissue for injection [12]. In the present application, the device was used to mechanically reduce the PRF fibrin matrix before incorporation with the harvested adipose tissue. The use of this device should not, however, be interpreted as establishing that micronization itself improves graft retention or clinical outcome.

The five-year clinical observation in this patient demonstrates sustained clinical contour correction following the overall procedure. Importantly, the durability of the result cannot be specifically attributed to PRF augmentation. Successful conventional structural fat grafting alone, the injection technique, recipient-site characteristics, postoperative management, and individual biological variability may all have contributed to the observed result. Because no untreated or conventionally grafted control site was available and no objective volumetric or imaging-based assessment was performed, the present case cannot distinguish the contribution of PRF from the effects of fat grafting itself.

The application of the technique to steroid-induced gluteal lipoatrophy is of particular technical interest because corticosteroid-associated soft tissue atrophy may produce localized volume loss accompanied by changes in the overlying skin and subcutaneous tissue [1-3]. Fat grafting can provide structural volume restoration in such settings, but the behavior of grafted fat in an altered recipient bed may be variable. The present case demonstrates that the described technique can be applied in such a setting and that sustained clinical contour correction was observed over five years. It does not establish that PRF provides an incremental benefit in this patient population.

The possibility of delayed changes following fat transplantation should also be considered during patient counseling. Delayed hypertrophy or overgrowth of transplanted fat has previously been described in the treatment of steroid-induced atrophy [13]. Although no clinically apparent overcorrection or recurrent contour deformity was observed during the five-year follow-up in this case, longer-term behavior may vary between patients.

Gluteal fat grafting also requires particular attention to procedural safety. The relatively small volume used in this case and the office-based local anesthesia setting differ substantially from high-volume gluteal augmentation procedures, but these factors do not eliminate the importance of appropriate injection-plane selection. Subcutaneous-only injection is an important safety principle intended to reduce the risk of deeper injection and pulmonary fat embolism [14]. In the present case, all graft placement was strictly confined to the subcutaneous plane, with no intramuscular or deeper-plane injection.

The present report also does not establish an optimal PRF preparation protocol or PRF-to-fat mixing ratio. Prior experimental work has investigated the effects of PRF mixing ratios on fat graft survival, but the optimal ratio may depend on the specific preparation technique, PRF characteristics, centrifugation parameters, and recipient environment [8]. The ratio used in the present case was 1:3 by volume (5 mL PRF to 15 mL adipose tissue) and was not prospectively optimized. Further investigation is required before a specific ratio can be considered optimal.

Limitations

Several limitations should be considered when interpreting this report. First, this is a single-patient technical report and therefore cannot establish the efficacy or generalizability of PRF-enhanced fat grafting. Second, there was no control group receiving conventional fat grafting alone, which prevents isolation of any potential contribution from PRF. The durable contour observed at five-year follow-up may reflect the effects of conventional structural fat grafting, the injection technique, recipient-site characteristics, postoperative management, or individual biological variability rather than PRF specifically.

Third, no objective volumetric measurement or imaging-based assessment was performed to quantify graft retention over time. Accordingly, the postoperative photographs provide clinical documentation of contour appearance but should not be interpreted as quantitative evidence of volumetric retention. Fourth, the available clinical photographs were not obtained under fully standardized conditions with respect to positioning, framing, lighting, clothing, and camera angle, which limits direct visual comparison across time points. Fifth, no stromal vascular fraction analysis or other cellular characterization was performed in this patient. Finally, the centrifuge model and rotor characteristics were not recorded for this historical procedure, preventing calculation of the relative centrifugal force (RCF); therefore, the centrifugation protocol is reported as 2800 rpm for five minutes. The PRF-to-fat ratio was defined for this case but was not prospectively optimized.

Future research directions

Future studies should compare PRF-enhanced fat grafting with conventional fat grafting alone using standardized PRF preparation protocols, predefined PRF-to-fat ratios, objective volumetric measurements, and appropriate imaging-based assessments. Such studies would be necessary to determine whether PRF provides a clinically meaningful incremental benefit in graft retention, contour durability, or tissue quality.

Although the described technique may warrant investigation in other localized contour deformities involving compromised soft tissue, extrapolation beyond the present case should be made cautiously. The current report provides evidence of technical feasibility and long-term clinical follow-up in a single patient rather than evidence of comparative efficacy.

## Conclusions

This report demonstrates the technical feasibility of incorporating mechanically micronized PRF into autologous structural fat grafting for a localized gluteal contour deformity in a compromised soft tissue bed. The patient demonstrated sustained clinical contour correction over five years of follow-up. However, the single-patient design, absence of a control group, and lack of objective volumetric or imaging-based assessment preclude determining whether PRF contributed to graft retention or tissue quality beyond the effects of fat grafting alone. Controlled studies with standardized PRF preparation, defined mixing ratios, and objective outcome assessment are needed to determine the efficacy and clinical utility of PRF augmentation.
